# Highly Cytotoxic Copper(II) Mixed-Ligand Quinolinonato Complexes: Pharmacokinetic Properties and Interactions with Drug Metabolizing Cytochromes P450

**DOI:** 10.3390/pharmaceutics15041314

**Published:** 2023-04-21

**Authors:** Martina Medvedíková, Václav Ranc, Ján Vančo, Zdeněk Trávníček, Pavel Anzenbacher

**Affiliations:** 1 Department of Pharmacology, Faculty of Medicine and Dentistry, Palacký University in Olomouc, Hněvotínská 3, 779 00 Olomouc, Czech Republic; 2 Institute of Molecular and Translation Medicine, Faculty of Medicine and Dentistry, Palacký University in Olomouc, Hněvotínská 5, 779 00 Olomouc, Czech Republic; 3 Regional Centre of Advanced Technologies and Materials (RCPTM), Czech Advanced Technology and Research Institute (CATRIN), Palacký University in Olomouc, Šlechtitelů 27, 779 00 Olomouc, Czech Republic

**Keywords:** copper(II) complexes, quinolinonato derivatives, cytochrome P450, isothermal titration calorimetry

## Abstract

The effects of two anticancer active copper(II) mixed-ligand complexes of the type [Cu(qui)(mphen)]Y·H_2_O, where Hqui = 2-phenyl-3-hydroxy- 1H-quinolin-4-one, mphen = bathophenanthroline, and Y = NO_3_ (complex **1**) or BF_4_ (complex **2**) on the activities of different isoenzymes of cytochrome P450 (CYP) have been evaluated. The screening revealed significant inhibitory effects of the complexes on CYP3A4/5 (IC_50_ values were 2.46 and 4.88 μM), CYP2C9 (IC_50_ values were 16.34 and 37.25 μM), and CYP2C19 (IC_50_ values were 61.21 and 77.07 μM). Further, the analysis of mechanisms of action uncovered a non-competitive type of inhibition for both the studied compounds. Consequent studies of pharmacokinetic properties proved good stability of both the complexes in phosphate buffer saline (>96% stability) and human plasma (>91% stability) after 2 h of incubation. Both compounds are moderately metabolised by human liver microsomes (<30% after 1 h of incubation), and over 90% of the complexes bind to plasma proteins. The obtained results showed the potential of complexes **1** and **2** to interact with major metabolic pathways of drugs and, as a consequence of this finding, their apparent incompatibility in combination therapy with most chemotherapeutic agents.

## 1. Introduction

Metal complexes are in the spotlight for many researchers worldwide due to their interesting biological properties. This interest has been strongly encouraged by the ground-breaking discovery of the antiproliferative properties of cisplatin [*cis*–diamminedichloridoplatinum(II), CDDP] [1] and further fuelled by the following discoveries of other platinum-based drugs, such as carboplatin, oxaliplatin, or satraplatin. Unfortunately, the platinum-based drugs possess a number of adverse effects, leading to leukopenia and neutropenia, thrombocytopenia and anaemia, hepatotoxicity, ototoxicity, cardiotoxicity, nausea and vomiting, diarrhoea, stomatitis, pain, alopecia, and cachexia [2,3]. Therefore, the primary goal of the research in the field of novel metal–based coordination compounds is to produce compounds with a better antitumor activity profile than those used in clinics while having much fewer negative side effects and, importantly, being able to overcome a considerable resistance of many types of cancer cells towards the applied platinum-based drugs [4].

In the past decade, many transition metal-based compounds have been synthesised and studied. Besides platinum-based complexes, the compounds containing iron, copper, cobalt, gold, ruthenium, or vanadium central atoms, e.g., Ferrocifen (for Fe) [5], Casiopeína IIIia (for Cu) [6], Co(III) alkyne or Co(III) Schiff base complexes (for Co) [7], Auranofin (for Au) [8], NAMI-A, KP1019, KP1339, and TLD 1433 (for Ru) [9], Vanadocenes (for V) [10] have shown promising anticancer results in numerous preclinical or clinical studies [11,12]. Moreover, the potential of transition metal complexes related to various biological activities has been reviewed in many papers up to now. The following papers belong among the most current ones: e.g., a review regarding cobalt Schiff base complexes as anticancer agents [13], N-heterocyclic carbene complexes of silver as anticancer agents [14], cytotoxic activities of Pt, Pd, Ru, and Au complexes [15], and metal complexes of 8-hydroxyquinoline with antileukemia activities [16].

**Figure 1:**
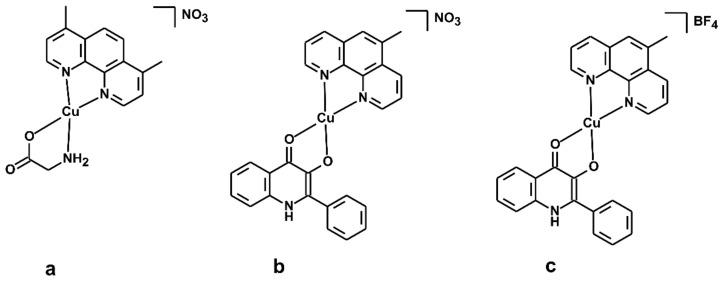
Structural formulas of one example from Casiopeínas^®^ family (**a**) together with the herein studied copper(II) complexes—complex **1** (**b**) and complex **2** (**c**).

Copper is one of the essential trace elements involved in cellular respiration, neurotransmission, and cellular metabolism [17,18,19]. Elevated levels of copper have been found in many types of human cancers, including prostate, breast, colon, lung, and brain. An overview of the chemistry, antitumor properties, and molecular targets of copper complexes on different types of cancer cells is described in a review article by Balsa et al. [20]. Copper is also essential for the function of several enzymes and proteins involved in energy metabolism, respiration, and DNA synthesis, notably cytochrome oxidase, superoxide dismutase (SOD), ascorbate oxidase, and tyrosinase. In fact, several copper chelators, targeting the copper ions in cancer cells, have been developed and are recently in the initial stages of clinical trials [21]. Considering the positive effects of copper on human organisms, many copper(II) complexes have been recently synthesised and tested on in vitro and in vivo antitumor activity [22]. A new family of copper-chelating compounds was designed and registered with the generic name Casiopeínas [23] (Figure 1a). The two general formulae of such compounds are [Cu(N-N)(N-O)]NO_3_ or [Cu(N-N)(O-O)]NO_3_, where the N-N donor ligand represents a polycyclic substituted diimine (e.g., 1,10-phenanthroline (*phen*) or 2,2′-bipyridine (*bpy*)), N-O indicates α-aminoacidate or a peptide, and the O-O donor ligand represents acetylacetonate (*acac*) or salicylaldehydate (*salal*) [24,25]. Several copper-based compounds of this new family demonstrated a promisingly high antiproliferative potency, compared with generally used cisplatin against human ovarian carcinoma, murine leukaemia (L1210), and other cell lines [26]. This fact started an intensive investigation of the anticancer properties of a wide range of copper complexes, and therefore, we have previously focused on the study of Casiopeínas^®^-like complexes containing bidentate *N*-donor heterocyclic ligands, such as 1,10-phenanthroline (*phen*) or 2,2′-bipyridine (*bpy*) or their derivatives herein abbreviated as N-N, and 2-phenyl-3-hydroxy-1H-quinolin-4-one and its derivatives (Hqui) of the general composition [Cu(N-N)(qui)]X·H_2_O (where X = NO_3_^−^ or BF_4_^−^) [27,28,29] (Figure 1b,c). The set of complexes exhibited a significant cytotoxic effect (with the IC_50_ values lying in the micromolar and sub-micromolar concentrations) against a broad spectrum of human cancer cell lines (A549, HeLa, G361, A2780, A2780cis, LNCaP, and THP-1) as compared with cisplatin [29]. Moreover, the in vitro toxicity of these complexes against healthy cells of primary human hepatocytes is significantly lower than that for human cancer cells, pointing out the relative high selectivity and safety of such complexes. This conclusion can also be supported by other findings related to the Cu(II)-casiopeinas-like complexes with the {CuN_2_O_2_} chromophore in the vicinity of the copper(II) atom originating from our group, which showed low in vitro toxicity against normal human hepatocytes, with IC_50_ > 100 μM [30,31].

In the previous publications by Buchtík et al. [27,28], it has been shown that the studied copper(II) complexes possess besides the considerable cytotoxicity against cancer cells also the ability to interact with DNA and human serum albumin in vitro and to promote oxidative damage towards the DNA molecule in vitro. Additionally, the complexes were able to interact with sulphur-containing biomolecules, such as reduced glutathione and L-cysteine, and thus they might act as both oxidative stress-promoting and antioxidant-depleting agents. The aforementioned molecular mechanisms have been associated with the induction of several cellular pathways leading to autophagy, apoptosis, and necrosis in cancer cells [32], or with a combination of these processes for which the new term “cuproptosis” [33] has been proposed. Recently, several molecular mechanisms of anticancer effects of heteroleptic copper(II) complex involving a quinoline-derived ligand were described [34], based on the induction of autophagy by activating the MAPK signaling pathway and induction of apoptosis via mitochondrial/ROS/ER-stress responses, i.e., the disruption of mitochondrial membrane potential, the induction of oxidative stress by overproduction of ROS and overexpression of CCAAT-enhancer-binding homologous proteins (CHOP), and the activation of eukaryotic initiation factor-2 (eIF2α) and protein kinase RNA-like endoplasmic reticulum kinase (PERK) in the A549 cells. The induction of autophagy and apoptosis through ROS generation and Jnk activation has also been described in the case of the copper(II) complex Cas III-ia from the Casiopeínas^®^ family, structurally similar to the title complexes [35]. Moreover, another member of the Casiopeínas^®^ family, known as Cas II-gly, showed, in addition to the significant antitumor effects, the ability to inhibit the activity of key energy metabolism enzymatic systems [36] (i.e., inhibited hexokinase and glycolysis and caused diminution in the ATP content, indicating inhibition of oxidative phosphorylation) in hepatocarcinoma AS-30D cells.

Since their discovery, a variety of analogous compounds with copper as the central atom have been synthesised, but there is still not enough information regarding their pharmacology properties, including interactions with the family of cytochromes P450 (hence P450s or CYP). Cytochromes P450 constitute a superfamily of heme enzymes and have been identified in single-cell organisms (such as bacteria) as well as higher organisms (e.g., humans). There are more than 2700 known isoenzymes of CYPs, and many of them are involved in the biotransformation of various natural compounds (e.g., fatty acids, steroids, and vitamin D) and xenobiotics (drugs, environmental toxins, food preservatives, and other foreign agents). Human drug-metabolising P450 enzymes are present in many sites in the human body, including the liver, gastrointestinal tract, brain, lungs, kidneys, and heart [37]. The 57 human P450 genes are primarily associated with phase I of drug metabolism, where the polar group is incorporated into the original substrate. The proteins of three CYP families, namely CYP1, CYP2, and CYP3, have important and unique functions due to their involvement in the first phase of biotransformation. In the current paper, we studied the fundamental pharmacological properties of the two selected complexes (shown in Figure 1b,c), including stability in plasma, chemical stability, binding affinity to proteins, and transport over an artificial membrane. Moreover, we studied the influence of these complexes on the enzymatic activity of nine human liver microsomal CYP forms, namely CYP1A2, CYP2A6, CYP2B6, CYP2C8, CYP2C9, CYP2C19, CYP2D6, CYP2E1, and CYP3A. The main reason why these two copper(II) complexes were chosen for the present study is related to the fact that they revealed positive cellular responses in human tumour cells (A549, HeLa, G361, A2780, A2780R, LNCaP, THP-1, HOS, and MCF-7) and relatively low cytotoxicity to healthy human hepatic cells [19,20,21]. One of the side goals of the study was also to discover if the presence of different counterions (i.e., NO_3_^−^ vs. BF_4_^−^) within the structures of the studied complexes could have any impact on the pharmacological, pharmacokinetic, and enzymatic features.

The study of new mixed-ligand transition metal complexes in order to develop a more efficient family of drugs able to fight threatening diseases, including cancer, is one of the most demanding tasks of contemporary medicine. The study of their interactions with cytochromes P450 presents one of the important steps in their development, especially due to the key roles of cytochromes P450 in the metabolism of many xenobiotics, including medications. That is one of the main reasons why we decided to realise this work.

## 2. Materials and Methods

The synthesis and thorough characterization of copper(II) mixed-ligand quinolinonato complexes, [Cu(qui)(mphen)]Y·H_2_O, where Hqui = 2-phenyl-3-hydroxy-1H-quinolin-4-one, mphen = 5-methyl-1,10-phenanthroline (bathophenanthroline), and Y = NO_3_ (1) (Figure 1b) or BF_4_ (2) (Figure 1c), including the analytical data regarding the purity of the compounds, were described previously in the literature [27,28]. The copper(II) complexes used in this work were obtained from the same batch as described in the literature [27,28]. Thus, the final formulas of the studied complexes were already confirmed and well known based on thorough characterization of the compounds.

Free 5-methyl-1,10-phenanthroline ligand and copper(II) nitrate trihydrate, used in this work for reference purposes, were purchased from Sigma-Aldrich (Prague, Czech Republic), and used as received. Cryopreserved pooled human liver microsomes were obtained from Xenotech (Lenexa, KS, USA), and human plasma was obtained from the Transfusion Department of University Hospital Olomouc (Olomouc, Czech Republic). Recombinant human cytochrome P450 3A4 was purchased from Sigma-Aldrich (Prague, Czech Republic), bactosomes CYP1A2 and CYP2A6 were obtained from BD Biosciences (Franklin Lakes, NJ, USA), and CYP3A4 from Cypex (Dundee, UK).

For the determination of CYP activities, ethoxyresorufin and 7-ethoxy-4-(trifluoromethyl)coumarin were purchased from Fluka (Buchs, Switzerland). Coumarin, testosterone, diclofenac, bufuralol, and chlorzoxazone were obtained from Sigma-Aldrich (Prague, Czech Republic); midazolam was purchased from Abcam (Cambridge, UK); paclitaxel was purchased from Chemos CZ (Prague, Czech Republic); and (*S*)-mephenytoin was purchased from Santa Cruz Biotechnology Inc. (Heidelberg, Germany).

Dimethyl sulfoxide (DMSO) and potassium dihydrogen phosphate were obtained from Lach-Ner (Neratovice, Czech Republic); dichloromethane, methanol, and acetonitrile were from VWR Prolabo (Fontenay-sous-Bois, France). All other chemicals were supplied by Sigma Aldrich CZ (Prague, Czech Republic).

### 2.1. In Vitro Pharmacological Properties

Pharmacological properties of the complexes **1** and **2**, including stability in plasma, microsomal stability, binding to plasma proteins, and permeability through an artificial membrane, were tested under in vitro conditions and according to methods described in previous reports [38,39]. Analysis of the samples was performed using Agilent RapidFire 300 High-Throughput Mass Spectrometry System (Agilent Technologies, Wakefield, MA, USA) with subsequent detection using the mass spectrometer Qtrap 5500 (AB Sciex, Concord, Toronto, ON, Canada), abbreviation RF-MS. The samples were aspirated directly from the plates into a 10 μL sample loop and passed through a C4 cartridge with solvent A (5% acetonitrile, 95% water, 0.1% formic acid) at a flow rate of 1.5 mL/min for 3 s. After the desalting step, analytes retained on the cartridge were eluted with solvent B (95% acetonitrile, 5% water, 0.1% formic acid) in the mass spectrometer at a flow rate of 0.4 mL/min for 5 s. The mass spectrometry of the samples was carried out using electrospray ionisation in the positive ion mode. Daughter ion peaks were identified using a multiple-reaction monitoring protocol.

### 2.2. Chemical Stability (Stability in PBS) and Stability in Human Plasma

The studied compounds (final incubation concentrations of 7.4 µM or 3.3 μM, respectively; DMSO did not exceed 0.1%) were incubated with 1 × PBS, pH 7.4, or preheated human plasma, respectively, at five different time points (0, 15, 30, 60, and 120 min) at 37 °C. The reaction was terminated by ice-cold methanol (chemical stability) or acetonitrile:methanol (2:1), and samples were left at −80 °C overnight (plasma stability). The solution was mixed and centrifuged (4000 rpm, 9 min, 4 °C). After lyophilization of supernatant, samples were dissolved in mobile phase with internal standard and analysed by RF/MS analysis.

### 2.3. Microsomal Stability Assay

According to the protocol, reaction mixtures included tested compound (5.74 μM), human liver microsomes, NADPH-generating system consisting of NADP^+^, isocitrate dehydrogenase, isocitric acid, and MgSO_4_ in 100 mM K/PO_4_ buffer [40]. Assay was performed at 0, 15, 30, and 60 min. Reactions were terminated using acetonitrile–methanol (2:1) mixture. After centrifugation, the supernatant was lyophilized and analysed by RF-MS. The values of intrinsic clearance were calculated using the formula: CL_int_ = 0.693/t_1/2_/mg microsome protein per mL of reaction.

### 2.4. Plasma Protein Binding (PPB)

The fraction bound value was determined using the semipermeable membrane that separates a protein-containing compartment (R chamber) from a protein-free compartment (W chamber) (Rapid Equilibrium Dialysis, Thermo Scientific™, Rockford, IL, USA) [41]. Into the chamber R was added 10 μM solution of the test compound in 50% plasma, and into the chamber W was added PBS buffer. After incubation (37 °C, 4 h), to the protein-free solution was added the same amount of 50% plasma, and to the protein-containing solution was added the buffer. The reaction was terminated by the addition of acetonitrile and methanol (2:1). Following centrifugation, the supernatant was lyophilized. Samples were dissolved in mobile phase containing internal standard and analysed on the RF/MS.

### 2.5. Parallel Artificial Membrane Permeability Assay (PAMPA)

PAMPA was performed with the Millipore MultiScreen filter MultiScreen-IP Durapore 0.45-μm plates and receiver plates (Merck Millipore, Burlington, MA, USA). The test compounds (20 μM in PBS) were added to the donor compartments, and the acceptor wells were filled with PBS (pH 7.4). The permeation of compounds across a filter membrane (activation by 10% lecithin in dodecane) was quantified by RF/MS after 18 h incubation at room temperature [42]. The apparent permeability, logPe, was calculated from the following equation: logPe = log{C× − ln(1 − drug_A_/drug_E_)}; C = (V_A_ × V_D_)/{(V_D_ + V_A_) × A × T}, where V_D_ and V_A_ are the volumes of the donor and acceptor compartments, respectively; A is the active surface area of the membrane; T is the time of the incubation in seconds; and drug_A_ or drug_E_ represents the concentration of the tested compounds in the acceptor compartment and in the solution in theoretical equilibrium.

### 2.6. Cytochrome P450 Activities

For each enzyme assay, the preliminary experiments to determine Michaelis constant (Km) and limiting velocity (Vmax) were performed to calculate the time of incubation (the specified time for which the reaction is still linear) and the values of substrate concentration (in the range corresponding to the value of Km). The amount of human liver microsomes (HLM; expressed as the amount of CYP in pmol and the concentration of HLM protein in mg/mL in the reaction vessel) in the reaction was determined using previously established protocols [40].

The reaction mixtures were buffered by 0.1 M potassium phosphate buffer (pH 7.4) and contained a NADPH-generating system consisting of the isocitrate dehydrogenase (6 U/mL), NADP^+^ (0.5 mM), isocitric acid (4 mM), and MgSO_4_ (5 mM). The amounts of human liver microsomes and concentrations of individual substrates in reaction mixtures are shown in Table 1. The final concentration levels of complexes were obtained by diluting a 10 mM solution with dimethyl sulfoxide (DMSO). It is known that DMSO can in some cases inhibit P450; the following measures were thus applied: (a) control samples containing organic solvent without the tested compounds were prepared and used as references; (b) DMSO levels were maintained below 0.1% to minimise the inhibition. Inhibition experiments were performed using five concentration levels of the tested compounds in the range of 10–100 µM. The maximum concentration was chosen so that in the case of inhibition, it would be possible to determine the inhibitory effect of the complexes and characterise them by determining the IC_50_ value. Additionally, an inhibitor-free control was used as a reference. Incubations were executed in two independent batches, and the samples were prepared and measured in triplicate at 37 °C. Analyses of the metabolites formed from the specific substrates were performed by HPLC using the Prominence system (Shimadzu, Kyoto, Japan) equipped with a LiChroCART 250-4 LiChrospher 100 RP-18 column, Chromolith^®^ HighResolution RP-18 end-capped column (Merck, Darmstadt, Germany), or Kinetex Biphenyl column (Phenomenex, Torrace, CA, USA).

Inhibition of individual activities of selected CYPs by the studied complexes, free ligands Hqui and mphen, and copper(II) nitrate trihydrate were in all cases evaluated by plotting the respective remaining activity against the concentration of the inhibitor. The apparent Ki values were determined by additional measurements using substrate concentrations corresponding to 1/2 Km, Km, 2 Km, and 4 Km in the case of inhibition. The type of model was selected on the basis of the Lineweaver–Burk and Dixon plots. The values of IC_50_ were obtained by analysing the plot of the percentage of activity remaining after inhibition versus the logarithm of the inhibitor concentration using the GraphPad Prism 6 software (La Jolla, CA, USA).

In addition to the above-described inhibition studies, a series of Single Point Assays was performed in order to uncover possible slow-binding or irreversible inhibition, which might be associated with the oxidative damage of the proteins. One half of the samples, containing the HLMs (in a 10-fold higher concentration as compared with normal activity experiments) and 25 µM concentration of the corresponding complex, or 1% DMSO representing the solvent control sample, were preincubated together with the NADPH-generating system (consisting of the isocitrate dehydrogenase (6 U/mL), NADP^+^ (0.5 mM), isocitric acid (4 mM), and MgSO_4_ (5 mM)) at 37 °C for 30 min. In the second half of the samples, only HLMs and corresponding complexes, or 1% DMSO, representing the solvent control sample, were preincubated at 37 °C for 30 min, and afterwards, the NADPH was added to the samples. Subsequently, an aliquot of each sample was taken, diluted 10-fold, and a specific substrate (at 5 km concentration) was added. The residual microsomal activity was measured as described above and compared with the activity of uninhibited microsomes. If the time-dependent inhibition occurs, then a decrease in activity should be visible only in the samples where the NADPH-generating system was present during the preincubation. To exclude the possible inhibitory effects of the solvent, the appropriate solvent reference samples were analysed, and no significant differences in the inhibition of the microsomal activities were observed among the two systems or the control (uninhibited) samples.

### 2.7. Spectroscopic Study of Interactions of the Studied Complexes with Human Liver Microsomes

Difference spectra analyses of the studied complexes, free ligands Hqui and mphen, and copper(II) nitrate trihydrate with human liver microsomes were performed according to a previously established protocol [43]. The cuvette contained microsomes diluted to final concentration of CYP 1 µM in 50 mM potassium phosphate buffer (pH 7.4). The tested compounds were dissolved in the same buffer, and their concentrations in the experiments ranged from 0.002 to 33.310 µM. The UV-Visible spectra were recorded at room temperature by repetitive scanning between 300 and 700 nm using Varian 500 UV/VIS spectrophotometer (Agilent Technologies, Wakefield, MA, USA). Changes of absorbance in the range of the Soret’s region (380–450 nm) were plotted against the concentration of the studied compounds. Data were analysed using GraphPad Prism 6 software (La Jolla, CA, USA).

### 2.8. Isothermal Titration Calorimetry (ITC)

The interactions of the compounds with selected forms of cytochrome P450, i.e., human bactosomes CYP1A2, CYP2A6, CYP3A4, and recombinant human CYP3A4, were studied using isothermal titration calorimetry (ITC). ITC experiments were performed at 25 °C with a Nano ITC Low Volume (TA Instruments, New Castle, DE, USA). During all measurements, 20 injections of 16µM of the studied compound (2.5 µL each) were titrated into 250 µL of protein (2 µM) with time intervals of 300 s and a stirring speed of 250 rpm. All ITC experiments were conducted with degassed buffered solutions (100 mM phosphate buffer, pH 7.4) in the presence of 1% DMSO. Control experiments included the titration of each complex solution into a buffer. The corrected data refer to experimental data after subtraction of the buffer control data from the compounds data. The resulting thermograms were analysed using the “Independent” model within the NanoAnalyze software (TA Instruments, New Castle, DE, USA).

### 2.9. DFT Geometry Optimisation (Computational Details)

Geometry optimisations of the [Cu(mphen)(qui)]^+^ complex cation were performed with the Spartan’14 (ver. 1.1.4) software package [44] using the density functional theory (DFT) with different functionals (B3LYP, BP, or ϖB97X-D) and in combination with LANZ2DZ, LACVP, or LACVP** basis sets. The calculations were performed in a vacuum.

## 3. Results and Discussion

### 3.1. Geometry Optimisation of the [Cu(mphen)(qui)]^+^ Cation

**Figure 2:**
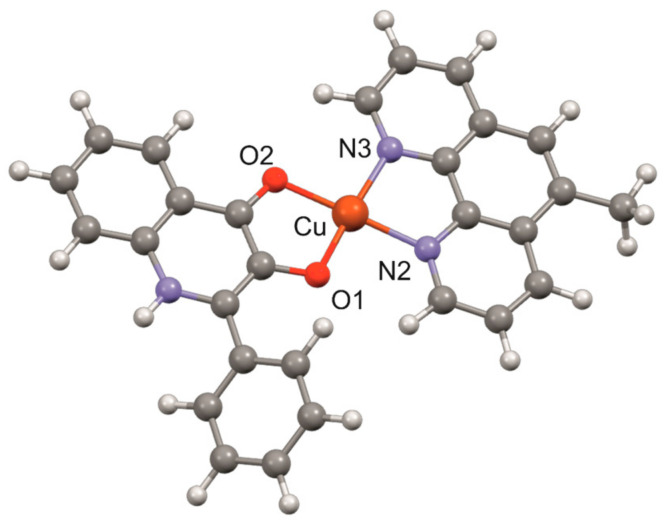
The geometry of the [Cu(mphen)(qui)]^+^ complex cation optimised at the ϖB97X-D/LACVP** level of theory.

Based on the fact that single crystal X-ray data are lacking for the studied complexes, we decided to predict the geometry of the complex cations of complexes **1** and **2** by means of quantum chemical calculations related to the geometry following from the X-ray structure of [Cu(qui)(phen)]NO_3_·H_2_O, which revealed the ionic nature of the nitrate anion, and the water molecule stays uncoordinated and behaves as a crystal solvent molecule [27]. The geometry of the [Cu(mphen)(qui)]^+^ species was optimised with the help of the Spartan’14 (Ver. 1.1.4) software [44] using density functional theory (DFT) involving different hybrid (B3LYP, BP, and ϖB97X-D) functionals with the LANL2DZ, LACVP, or LACVP** basis sets. The optimised geometry of the cation is shown in Figure 2. The copper(II) atom is coordinated by two nitrogen atoms from the mphen ligand and by two oxygen atoms from the qui ligand in a distorted square-planar geometry. With the aim of finding a proper combination of the method and basis sets to determine geometry that would give realistic information regarding the geometry and interatomic parameters around the vicinity of the central copper(II) atom, combinations of different functionals and basis sets were employed. The results are summarised in Table 2, and they revealed that the ϖB97X-D/LACVP and ϖB97X-D/LACVP** levels of theory provided meaningful data with minimal discrepancies between calculated and experimentally determined data associated with the known X-ray structure for [Cu(phen)(qui)]NO_3_. The coordinates for the best-optimised geometry of the complex cation are given in Supplementary Materials (see Table S1) in the XMol XYZ format.

### 3.2. Pharmacokinetic Properties of the Studied Compounds

The study of absorption, distribution, metabolism, and excretion (ADME) presents an important stepping stone for the evaluation of the application potential of new pharmaceutical substances. At first, the chemical stability of the investigated copper-based complexes in human plasma, their binding to plasma proteins, their microsomal stability, and their permeability through an artificial membrane (PAMPA) were studied. All the tested compounds were found to be stable in PBS and human plasma after incubation for 2 h, where more than 91% of the original substances were detected in the intact forms. For more information, see Table 3. Additional experiments were performed in which the compounds were incubated in PBS and human plasma for 72 h, as is the case for the assessment of toxicity in cancer cell lines. Both complexes showed stability of about 95% of the control even after such a long time (see Figure S1 in Supplementary Materials). Metabolic stability in human liver microsomes was measured for 60 min. The remaining percentages of the intact forms were 78% for complex **1** and 70% for complex **2**, respectively. According to the tabulated values, these complexes fall into the category of having a medium metabolic rate, according to Nassar [45]. The binding of the studied compounds to plasma proteins influences primarily the distribution of the drug in the organism. The percentages of complex **1** and complex **2** binding to plasma proteins were 95% and 93%, respectively. The passive diffusion mechanism for both compounds through the artificial membrane was classified as low compared with the control compounds, propranolol and atenolol [46].

### 3.3. Effects of the Complexes and Free Hqui and mphen Ligands on Catalytic Activities Cytochrome P450 in Human Liver Microsomes

**Figure 3:**
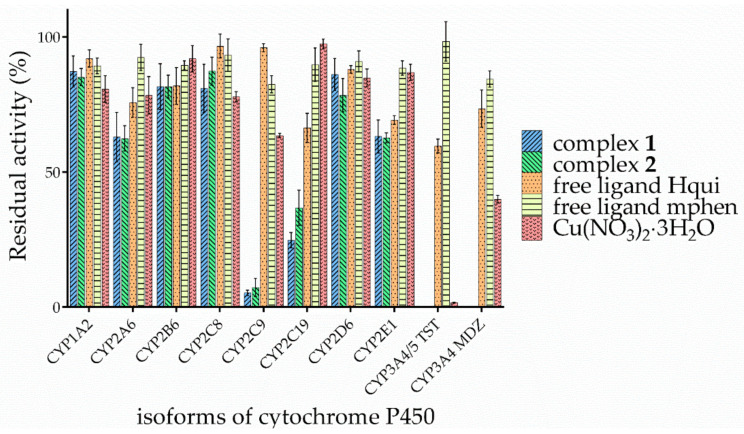
Overview of inhibition rates of CYP activities with specific substrates by the copper(II) complexes **1** and **2,** free Hqui and mphen ligands, and Cu(NO_3_)_2_·3H_2_O at 100 μM concentration. The results represent the amount of residual activity of CYPs expressed as a percentage of the activity of an uninhibited enzyme.

Next, we studied the influence of the complexes and the free Hqui (2-phenyl-3-hydroxy-1H-quinolin-4-one) and mphen (5-methyl-1,10-phenanthroline) ligands on the enzymatic activities of the nine isoforms of cytochrome P450, present in human liver microsomes. Specifically, the inhibition of activities of CYP1A2, CYP2A6, CYP2B6, CYP2C8, CYP2C9, CYP2C19, CYP2D6, CYP2E1, and CYP3A were evaluated. The tested compounds were studied at concentration levels of 0, 10, 25, 50, 75, and 100 μM. The activity of all the tested isoforms of P450 was found to be considerably influenced at the maximum concentration of the studied compounds (the detailed comparison is shown in Figure 3). The control experiments using the Hqui ligand (2-phenyl-3-hydroxy-1H-quinolin-4-one) and mphen ligand alone were elaborated in order to further evaluate the possible mechanism of action. Interestingly, only negligible interaction of the free ligands with CYP isoenzymes was detected, even at concentration levels approaching 100 μM. To some extent, the activity of CYP3A4/5 (which decreased to ca. 60%) was affected by the Hqui ligand. Moreover, both complexes clearly inhibited the enzymatic activities of all the studied isoforms of CYP, albeit with varying degrees of power, ranging from 10% inhibition for CYP2C8 to 99% inhibition for CYP3A4/5.

**Figure 4:**
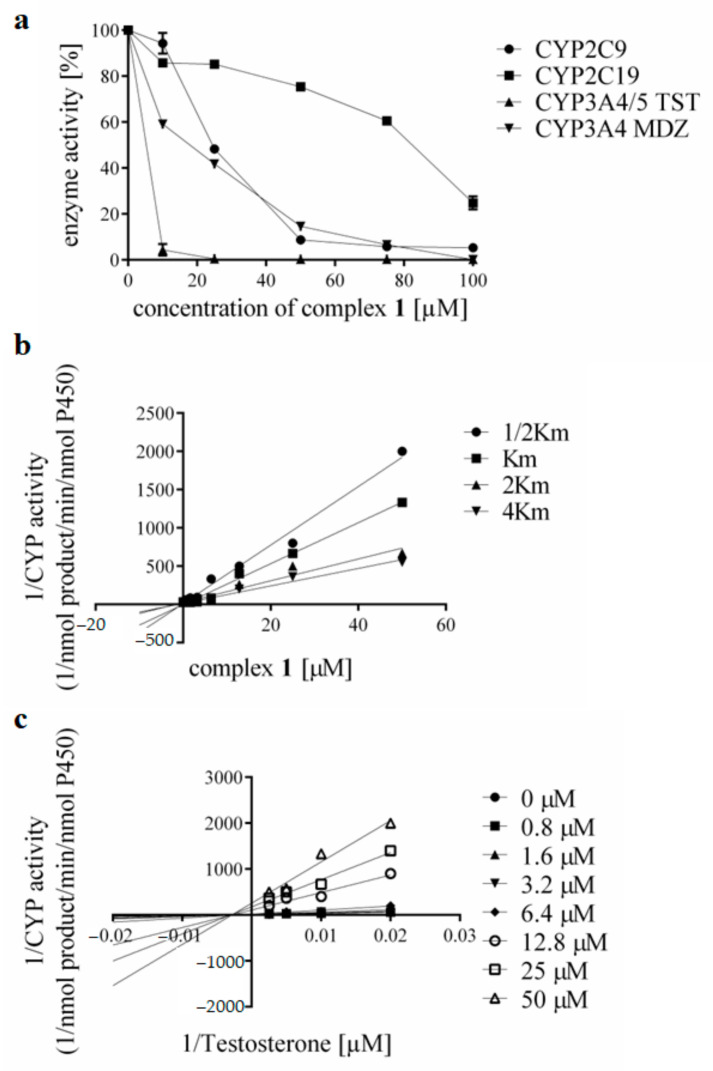
(**a**) The effect of complex **1** on the enzymatic activity of CYP2C9, CYP2C19, and CYP3A4/5 with the specific substrates of testosterone and midazolam in HLMs. The inhibition of activity is determined as the mean of the two independent experiments performed in triplicates ± SD and is expressed as a percentage of activity remaining relative to the control (set to 100%, without the addition of the studied compounds). Concentrations of complex **1** in reaction mixtures were 0, 10, 25, 50, 75, and 100 μM. (**b**) Dixon plot for inhibition of CYP3A4/5 (testosterone 6β-hydroxylation) by complex **1**, and (**c**) Lineweaver–Burk plot for inhibition of CYP3A4/5 (testosterone 6β-hydroxylation) enzymatic activity by complex **1** at four substrate concentrations (50, 100, 200, 400 µM) for eight concentrations of complex **1** (0, 0.8, 1.6, 3.2, 6.4, 12.8, 25 and 50 μM).

Both complexes considerably influence the enzymatic activities of CYP2C9 and CYPC19, which could potentially result in an unwanted alteration in the metabolism of several drugs, including warfarin [47,48]. Warfarin is metabolised by CYP2C9, where the inhibition by both compounds is around 90%. The CYP3A4/5 isoforms are a crucial endpoint in the evaluation of the inhibitory properties of potential drug candidates because they metabolise over 50% of all the drugs used in clinics. The severe inhibitions to 1% of the basal activity were observed in the case of the CYP3A4 isoenzymes in both the testosterone 6β-hydroxylation and midazolam-1′-hydroxylation assays. The mechanism of the action is still under investigation; however, it could be related to the specific structure of the active site. For more information, the impact of complex **1** on the activity of the selected and most important three drug-metabolising P450s is shown in more detail in Figure 4a. The effect of the studied compounds on the enzymatic activities of all isoforms of cytochrome P450 can be seen in detail in Figure S2 in Supplementary Materials.

The IC_50_ values for the studied P450s were: 16.34 ± 1.15 μM for CYP2C9, 61.21 ± 0.74 μM for CYP2C19, 2.46 ± 1.14 μM for CYP3A4/5 (using the testosterone 6β-hydroxylation assay), and 28.79 ± 2.55 μM for CYP3A4 (using the midazolam-1′-hydroxylation assay). A comparison of the IC_50_ of the complexes of the selected forms of cytochrome P450 is shown in Figure S3 in Supplementary Materials. Consequently, several experiments were performed to study the mechanism of inhibition and the influence of both complexes on the resulting Ki values. The Dixon and Lineweaver–Burk plots with four substrate concentrations (corresponding to ½ Km, Km, 2Km and 4Km) were constructed, and the results of the calculations are shown in Figure 4b,c. Complex **1** revealed a non-competitive type of inhibition for all three studied forms of cytochromes P450, with Ki values of 2.56 ± 0.37 μM for CYP2C9, 4.15 ± 0.71 μM for CYP2C19, and 3.60 ± 0.67 μM for CYP3A4/5 (testosterone 6β-hydroxylation assay). This finding can be interpreted as an inhibition of the product formation by the presence of an inhibitor. The inhibitor is bound to a site in close proximity to the substrate; however, the inhibition is not conditioned by a conformational change of the active site because of the previous binding of a substrate. In the case of complex **2**, at 100 μM concentration, the CYP2C9 activity was inhibited down to 7% of the control, with the IC_50_ calculated as 37.25 ± 1.26 μM. Activity of the CYP2C19 form was inhibited to 36% of the basal activity (IC_50_ was 77.07 ± 0.74 μM). The activity of the isoforms CYP3A4/5 was also significantly reduced to 1%, while the IC_50_ was calculated as 4.88 ± 1.05 μM for the testosterone 6β-hydroxylation assay and 32.98 ± 1.13 μM for the midazolam 1′-hydroxylation assay. Experimental data obtained with complex **2** were analogically analysed by Dixon and Lineweaver–Burk plots to evaluate the possible mechanisms of enzyme inhibition. The course of these plots indicates a fully non-competitive inhibition for forms CYP2C9, CYP2C19, and CYP3A4/5 with Ki values of 0.92 ± 0.25 μM, 9.14 ± 0.80 μM, and 3.94 ± 0.60 μM, respectively.

Interestingly, inhibition by the CYP activities of CYP2C9 and CYP3A4 enzymes is the most prominent, which is in line with earlier results on the properties of their active sites. CYP3A4 and CYP2C9 have been shown by molecular dynamics studies and absorption spectroscopy at high pressure to possess the most flexible active sites able to accommodate bulky ligands, as both the complexes exhibiting extensive inhibition of these forms of cytochrome P450 activities are [49].

In additional experiments, the effect of the Cu^2+^ ions (in the form of the solution of copper(II) nitrate trihydrate) on cytochrome P450 activities was determined with the aim to compare the degree of inhibition among the complexes, the free ligands (Hqui and mphen), and Cu(NO_3_)_2_·3H_2_O (see Figure 3). The results showed that Cu(NO_3_)_2_·3H_2_O has a greater effect on the activity of the CYP3A forms for both specific substrates (testosterone and midazolam). Subsequently, we determined the type of inhibition for these two forms by Dixon and Lineweaver–Burk plots with four substrate concentrations (corresponding to ½ km, km, 2 km, and 4 km) (see Figure S4 in Supplementary Materials). The copper(II) nitrate trihydrate revealed a non-competitive type of inhibition for both the studied forms of cytochromes P450, with Ki values of 1.56 ± 0.25 μM (testosterone 6β-hydroxylation assay) and 8.25 ± 1.64 μM (midazolam 1′-hydroxylation assay).

The obtained data indicate that the nitrate (NO_3_^−^) complex has a slightly higher effect on CYP450 activity compared with the tetrafluoroborate (BF_4_^−^) complex. This effect is more pronounced, especially in the most affected forms of cytochrome P450, namely CYP2C9, CYP2C19, and CYP3A4/5. The free Hqui ligand influenced the CYP3A activity, but its effect was not as significant as in the case of the individual studied complexes. It could also have had a slight effect on CYP2C19 activity, with a 66% decrease in activity. Although for almost every isoform of cytochrome P450, complex **1** showed slightly stronger inhibition than complex **2** (as evidenced by the comparison of IC_50_ values; see Figure S3 in Supplementary Materials), the impact of the presence of different counterions (NO_3_^−^ and BF_4_^−^) within the structures of the studied complexes was not significant. As compared with a structurally similar compound, Cas III-Ea, and [Cu(acac)(4,7-dimethyl-1,10-phenanthroline)(H_2_O)](NO_3_)_2_ with IC_50_ values around 10 μM [50], the studied complexes **1** and **2** were weaker inhibitors of cytochrome P450, The studied complexes differed also in the mode of inhibition, i.e., the studied complexes showed non-competitive types of inhibition, while the previously mentioned members of the Casiopeínas^®^ family showed strong irreversible competitive inhibition of cytochrome P450. The difference in the degree of inhibition when taking into account the counterions (i.e., between the complexes **1** and **2**) was at most 12% for CYP2C19, but for the other isoforms the differences were at most about 5%. In all cases, however, these differences were not statistically significant. No differences were observed between the effects of both studied complexes on the activities of CYP2A6, CYP2B6, and CYP2E1.

Further experiments were performed in order to exclude possible redox reactions or the formation of hydroxyl radicals due to the presence of NADPH. We analysed the studied complexes in individual time periods during 1 h in the presence of the NADPH-generating system (see Figure S5 in Supplementary Materials). The complexes show stability throughout the incubation, so it can be assumed that during the study of cytochrome P450 activity there is no reduction of compounds, formation of hydroxyl radicals, or other changes in the structure of the complexes.

As part of the basic testing of potential inhibitors, we also checked if there was no time-dependent inhibition, i.e., that the inhibitory potential of the compounds did not increase with increasing preincubation time, e.g., by the formation of an enzyme-binding product. The studied complexes were subjected to a Single Point Assay to investigate a possible time-dependent mechanism in which the test compound is preincubated in the presence and absence of NADPH with microsomes. The data showed that no time-dependent or irreversible inhibition was caused by the complexes or by the used solvent (see Figure S6 in Supplementary Materials).

### 3.4. Spectral Interaction Studies of the Complexes, Free Hqui and mphen Ligands, and Copper(II) Nitrate Trihydrate with Human Liver Microsomal CYPs

**Figure 5:**
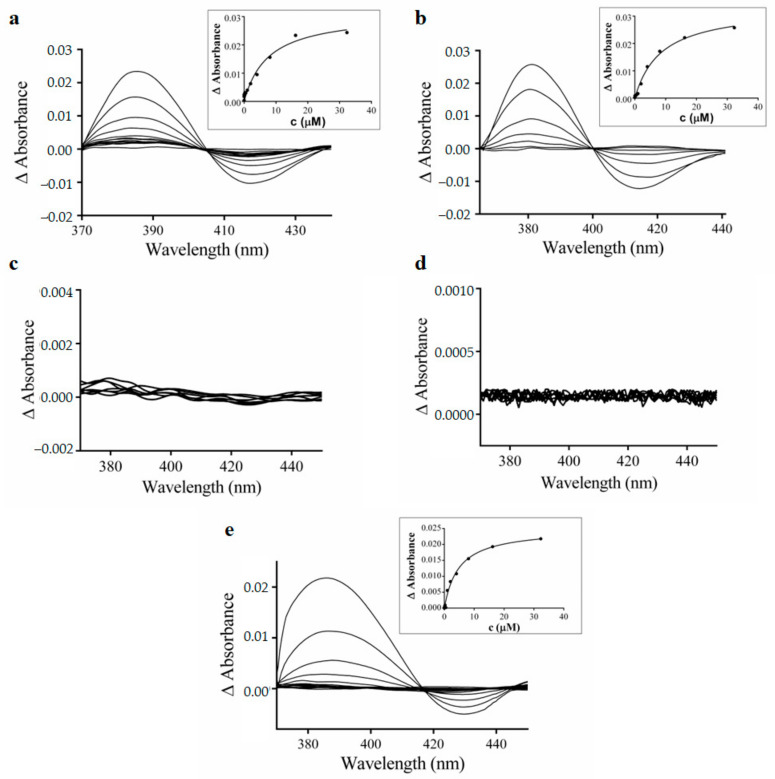
Difference spectra demonstrating the interaction of human liver microsomal cytochrome P450s with complex **1** (**a**), complex **2** (**b**), free Hqui ligand (**c**), free mphen ligand (**d**), and Cu(NO_3_)_2_·3H_2_O (**e**). CYPs concentration was 1 μM, and the concentrations of the tested complexes varied from 0.002 to 33.310 μM. Insets: plots of the absorbance changes at 386 nm vs. concentration of the respective compound.

In the next stage of the study, the interactions of the complexes with human liver microsomal CYPs were studied by means of UV-visible spectral titrations. The central atom of iron in the CYP heme enzymes could exist in two different spin states. The high-spin state is characterised by an absorption maximum at a wavelength of about 394 nm, and the low-spin state is characterised by an absorption maximum at a wavelength of about 417 nm in the UV-visible spectrum. Therefore, if the absorption maximum arises in the range of 410–420 nm, this indicates a direct interaction of the tested compound with the heme centre. On the other hand, the absorption maximum in the 380–400 nm region indicates the binding of the tested compound to other parts of the CYP protein. The binding of the studied compounds is characterised by the formation of an altered spectra with an absorption maximum shifted to 380–390 nm, which indicates binding of the tested complexes near the active site of CYP proteins. At the same time, the decreased absorption at about 417 nm occurred. The obtained results are shown in Figure 5a,b. The determined Ks constant expresses the substrate concentration, which forms half of the maximum spectroscopic change upon titration of CYP with a substrate. The Ks expresses the relative affinity of the substrates for the microsomal cytochromes P450 [51]. This value was determined from the hyperbolic yield curve constructed from the respective spectral differences at 386 nm versus the complex concentration. The Ks values for complex **1** and complex **2** were 7.59 ± 1.80 μM, and 8.56 ± 1.00 μM, respectively. The progress of the spectral changes in the absorption spectra in the maxima at 386 nm versus complex concentration is illustrated in the inserts in Figure 5. In contrast, no significant changes were observed in spectral binding studies involving the free ligands Hqui and mphen when interacting with microsomal CYPs (see Figure 5c–e, respectively). On the other hand, the copper nitrate trihydrate induced upon interaction with the CYP protein changes the differential spectra with an absorption maximum in the range of 380–390 nm and a minimum at 417 nm. The Ks value for Cu(NO_3_)_2_·3H_2_O was 9.81 ± 0.6 μM. There are no significant differences between the Ks values for both complexes **1** and **2** of copper nitrate trihydrate. These findings are in agreement with the results of inhibition studies of cytochrome P450 activities.

### 3.5. Thermodynamic Characterization of Interaction between Complexes and Cytochrome P450 Using Isothermal Titration Calorimetry (ITC)

**Figure 6:**
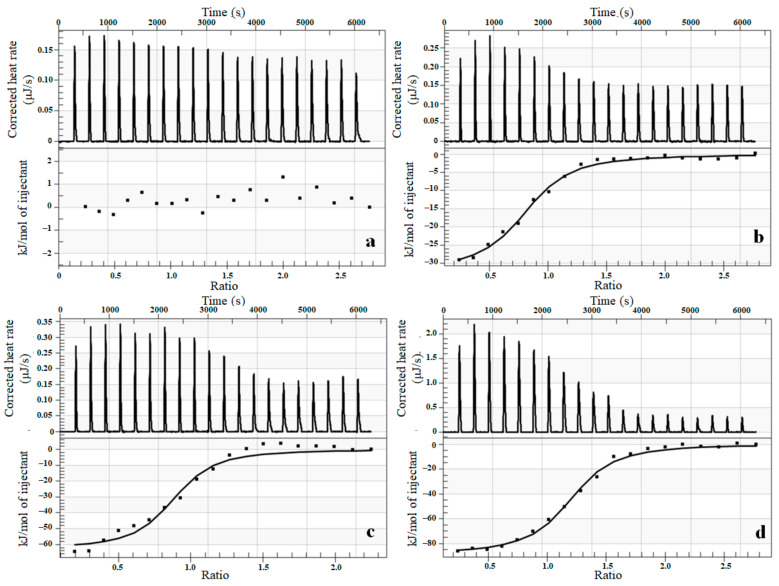
Representative ITC data for complex **1** binding to bactosomes CYP1A2 (**a**), bactosomes CYP2A6 (**b**), bactosomes CYP3A4 (**c**), and recombinant human CYP3A4 (**d**). (Top) Raw data plot of heat flow against time for the titration of CYP with complex **1**. (Bottom) Plot of molar enthalpy change against the complex **1**/cytochrome P450 molar ratio.

Finally, we utilised the ITC to analyse key thermodynamic parameters with relevance to the binding process of the complexes to cytochrome P450. The respective isoforms, namely human bactosomes CYP1A2, CYP2A6, CYP3A4, and recombinant human CYP3A4, were selected based on their susceptibility to inhibition by the studied complexes. Titration of all compounds into bactosomes containing CYP1A2 resulted in a weak exothermic effect, yet no significant complex-enzyme interaction was observed based on ITC titration results as shown in Figure 6a for complex **1**. The thermodynamic parameters for the interaction of tested compounds with CYP2A6 obtained from ITC are listed in Table 4. The obtained data indicate an exothermic reaction, as shown in Figure 6b. However, much stronger interactions were identified between the complexes and CYP3A4, which further supports our previous results based on the inhibition of CYP3A4 activity. The change in the measured enthalpy is approximately three times higher compared with results obtained on CYP2A6. Further, the thermodynamic parameters of the interaction with bactosomes expressing CYP3A4 and the recombinant human CYP3A4 were compared. First, the observed exothermicity of the calorimetry peaks originates from the strong interaction. As the sites available on enzymes become progressively occupied during the titration, the exothermicity of the peaks decreases and eventually saturates, similarly as previously demonstrated by Kastritis [52]. The measured parameters for this form of cytochrome P450 indicate that the interactions for both complexes are similar for bactosomes and the recombinant human protein. The representative calorimetric titration profiles of complex **1** with CYP3A4 are shown in Figure 6c,d, where the solid smooth line for complex **1** represents the best fit of the experimental data using the independent binding sites model. Summarised results can be seen in Table 4.

Both the studied compounds showed a binding ratio of complex enzymes (stoichiometry n) of approximately 1:1. The association constants Ka between the tested compounds and individual isoforms of cytochrome P450 were in the range of 10^6^–10^7^ M^−1^. The obtained values of ΔH were in all cases negative, showing that binding was exothermic. The change in the entropy level for the interaction with CYP2A6 was positive, which suggests a hydrophobic interaction on the surface of the protein. On the other hand, changes in the entropy levels were negative in the case of CYP3A4. This further suggests the presence of conformational changes and indicates that water molecules were released from the complex surface during the study, as proposed earlier by Jelesarov [53]. The negative ΔG values for all studied interactions suggest their spontaneous nature.

## 4. Conclusions

We investigated the basic pharmacological properties and possible effects on the activity of key isoforms of cytochrome P450 by two heteroleptic copper(II) complexes differing in the counterions (NO_3_^−^ versus BF_4_^−^), i.e., [Cu(qui)(mphen)]Y·H_2_O, where Y = NO_3_ (complex **1**) or BF_4_ (complex **2**). Both complexes significantly inhibited the activities of CYP2C9 (93% inhibition), CYP2C19 (70% inhibition), and more than 95% inhibition of CYP3A4 activity at a 100 µM concentration level. The presence of different counterions (NO_3_^−^ versus BF_4_^−^) within the structures of the studied complexes had no considerable impact on the activity of cytochrome P450. These findings correlate with the previously reported in vitro cytotoxicity against human cancer cells, where the differences between the complexes containing NO_3_^−^ and BF_4_^−^ counterions were negligible. Complex **2** has generally slightly lower inhibition effects towards the studied CYP isoenzymes, apart from CYP1A2 and CYP2D6. The results following from differential spectroscopy techniques show direct binding of both complexes to proteins. Finally, these studied compounds show several similarities in their pharmacokinetic behaviour. Both are moderately metabolised by human microsomes and are stable in plasma, where they also show high binding to plasma proteins. The transcellular permeation of the complexes was classified as low compared with other commonly used drugs in the parallel artificial membrane permeability assay (PAMPA), used as an in vitro model of passive diffusion.

## Figures and Tables

**Table 1:** Summary of the cytochrome P450 enzyme assay conditions.

CYP450 Form	CYP450 Activity Assays	CYP (pmol)	Substrate	Km (μM)	Incubation Time (min)
CYP1A2	7-Ethoxyresorufin *O*-deethylation	35	Ethoxyresorufin	1.56	15
CYP2A6	Coumarin 7-hydroxylation	35	Coumarin	14.00	15
CYP2B6	7-Ethoxy-4-trifluoromethylcoumarin 7-deethylation	35	7-Ethoxy-4-trifluoromethylcoumarin	15.25	15
CYP2C8	Paclitaxel 6-hydroxylation	70	Paclitaxel	18.41	15
CYP2C9	Diclofenac 4′-hydroxylation	35	Diclofenac	16.00	25
CYP2C19	(*S*)-Mephenytoin 4′-hydroxylation	50	(*S*)-Mephenytoin	28.00	25
CYP2D6	Bufuralol 1′-hydroxylation	70	Bufuralol	14.30	20
CYP2E1	Chlorzoxazone 6-hydroxylation	160	Chlorzoxazone	56.00	20
CYP3A4/5	Testosterone 6β-hydroxylation	100	Testosterone	100.00	20
CYP3A4	Midazolam 1′-hydroxylation	13	Midazolam	2.20	8

**Table 2:** A comparison of the selected interatomic parameters (in Å, °) within the [Cu(mphen)(qui)]^+^ complex cation obtained using DFT calculations at different levels of theory with those determined using a single crystal X-ray analysis of [Cu(phen)(qui)]NO_3_*.

	B3LYP		BP		ϖB97X-D			
	LANL2DZ	LACVP	LANL2DZ	LACVP	LANL2DZ	LACVP	LACVP**	X-ray *
Cu–O1	1.912	1.918	1.955	1.960	1.894	1.901	1.879	1.892(2)
Cu–O2	1.944	1.949	1.976	1.982	1.935	1.941	1.923	1.916(2)
Cu–N2	2.024	2.017	2.022	2.015	2.011	2.003	2.024	1.978(2)
Cu–N3	2.021	2.014	2.022	2.015	2.013	2.005	2.025	1.988(2)
O1–Cu–N3	175.76	175.86	166.48	166.66	176.00	176.10	175.52	176.22(8)
O2–Cu–N2	175.57	175.70	166.19	166.34	177.95	177.93	177.50	177.98(8)

* Data adopted from Buchtik et al. [27].

**Table 3:** Pharmacological parameters of the complexes **1** and **2**.

	% Compound Remaining							
	Chemical Stability				Plasma Stability			
Compound	15 min	30	60	120	15 min	30	60	120
complex **1**	99.6 ± 3.6	99.9 ± 1.5	97.8 ± 3.4	96.6 ± 3.4	98.9 ± 2.5	94.0 ± 3.3	94.2 ± 2.3	91.2 ± 1.2
complex **2**	99.6 ± 3.0	96.8 ± 2.4	94.0 ± 3.3	96.6 ± 2.4	98.3 ± 2.4	94.1 ± 2.4	96.6 ± 3.4	92.6 ± 2.2
	% Compound remaining							
	Microsomal stability			Plasma protein binding		PAMPA		
Compound	15 min	30	60	% Fraction bound		log P_app_	Category ^a^	
complex **1**	99.4 ± 3.5	80.7 ± 2.8	78.1 ± 1.7	95.1 ± 1.0		−8.93 ± 0.58	Low	
complex **2**	76.5 ± 2.7	76.2 ± 2.7	69.7 ± 2.4	92.9 ± 1.5		−8.69 ± 0.66	Low	

^a^ According to the log P_app_ obtained from the reference drugs, compounds with log P_app_ > −5 were categorised as highly permeable, while those with log P_app_ < −5 were considered poorly permeable [46].

**Table 4:** Thermodynamic parameters defining the interactions of complexes **1** and **2** with CYPs based on ITC titrations.

		Ka	ΔH	n	Kd	ΔS	ΔG
		(1/M)	(kJ/mol)		(nM)	(J/mol.K)	(J/mol)
complex **1**	CYP1A2B	no interaction					
	CYP2A6B	1.04 × 10^6^	−31.81	0.801	962.0	27.64	−40,050.9
	CYP3A4B	1.92 × 10^7^	−80.02	0.911	52.1	−129	−41,558.7
	CYP3A4	1.30 × 10^7^	−88.06	1.171	76.9	−178.3	−34,899.9
complex **2**	CYP1A2B	no interaction					
	CYP2A6B	3.12 × 10^6^	−23.04	1.071	321.0	47.06	−37,070.9
	CYP3A4B	2.17 × 10^7^	−62.71	0.859	46.1	−69.86	−41,881.2
	CYP3A4	1.59 × 10^7^	−51.77	1.095	62.9	−154.9	−5586.6

## Data Availability

Not applicable.

## Supplementary Materials

The following supporting information can be downloaded at: https://www.mdpi.com/article/10.3390/pharmaceutics15041314/s1, Table S1: The coordinates for the optimised geometry of the [Cu(mphen)(qui)]^+^ complex cation using the ϖB97X-D/LACVP level of theory in the XMol XYZ format; Figure S1: Stability of the tested complexes with plasma (**a**) and PBS (**b**). The stability was determined as the mean of the two independent experiments performed in triplicates ± SD and is expressed as a percentage of time remaining relative to the control (time 0). The studied compounds (final incubation concentration: 10 µM) were incubated with plasma and PBS at seven different time points (0, 30 min, 1, 2, 24, 48, 72 h) at 37 °C; Figure S2: The effect of complex **1** (**a**), complex **2** (**b**), free ligand Hqui (**c**) on enzymatic activity of all forms of cytochrome P450. Complementary experiments showing the effect of ligand mphen (**d**) and copper nitrate trihydrate (**e**) on enzymatic activity of CYP2C9, CYP2C19, and CYP3A with the specific substrates (Table 1 in the main text) in HLMs were performed. The inhibition of activity is determined as the mean of the two independent experiments performed in triplicates ± SD and is expressed as a percentage of activity remaining relative to the control (set to 100%, without the addition of the studied compounds). Concentrations of the complexes and ligands in reaction mixtures were 0, 10, 25, 50, 75, and 100 μM. TST = testosterone, MDZ = midazolam; Figure S3: Comparison of IC_50_ (**a**) and Ki (**b**) of the complexes **1** and **2** for inhibition of the selected forms of cytochrome P450; Figure S4: (**a**) Dixon plot for inhibition of CYP3A4/5 (testosterone 6β-hydroxylation) by Cu(NO_3_)_2_·3H_2_O and (**c**) for inhibition of CYP3A4 (midazolam 1′-hydroxylation) by Cu(NO_3_)_2_·3H_2_O, (**b**) Lineweaver–Burk plot for inhibition of CYP3A4/5 (testosterone 6β-hydroxylation) enzymatic activity by Cu(NO_3_)_2_·3H_2_O at four substrate concentrations (50, 100, 200, 400 µM) and (**d**) for inhibition of CYP3A4 (midazolam 1’-hydroxylation) by Cu(NO_3_)_2_·3H_2_O at four concentrations (1.1, 2.2, 4.4, 8.8 μM) for eight concentrations of Cu(NO_3_)_2_·3H_2_O (0, 0.8, 1.6, 3.2, 6.4, 12.8, 25 and 50 μM); Figure S5: Stability of the tested complexes with NADPH-generating system. The stability is determined as the mean of the two independent experiments performed in triplicate ± SD and is expressed as a percentage of remaining relative to the control (time 0). The studied compounds (the final incubation concentration was 10 µM) were incubated with NADPH-generating system at ten different time points (0, 5, 10, 15, 20, 25, 30, 35, 45, and 60 min.) at 37 °C; Figure S6: Single Point Assay of complexes **1** and **2**, and DMSO as a vehicle solvent in the presence or absence of NADPH. In one case, the tested complexes were preincubated in the presence of NADPH and microsomes at a concentration 10 times higher than in conventional activity testing. In the second case, NADPH was added to the sample containing microsomes and the compounds only after preincubation.

## References

[1] Rosenberg B., Van Camp L., Krigas T. (1965). Inhibition of Cell Division in Escherichia coli by Electrolysis Products from a Platinum Electrode. Nature.

[2] Johnstone T.C., Park G.Y., Lippard S.J.M. (2014). Understanding and Improving Platinum Anticancer Drugs—Phenanthriplatin. Anticancer Res..

[3] Wang D., Lippard S.J. (2005). Cellular processing of platinum anticancer drugs. Nat. Rev. Drug. Discov..

[4] Zhou J., Kang Y., Chen L., Wang H., Liu J., Zeng S. (2020). The Drug-Resistance Mechanisms of Five Platinum-Based Antitumor Agents. Front. Pharmacol..

[5] Jaouen G., Vessieres A., Top S. (2015). Ferrocifen type anticancer drugs. Chem. Soc. Rev..

[6] Tabti R., Tounsi N., Gaiddon C., Bentouhami E., Désaubry L. (2017). Progress in Copper Complexes as Anticancer Agents. Med. Chem..

[7] Munteanu C.R., Suntharalingam K. (2015). Advances in cobalt complexes as anticancer agents. Dalton Trans..

[8] Roder C., Thomson M.J. (2015). Auranofin: Repurposing an Old Drug for a Golden New Age. Drugs R&D.

[9] Pragti K., Bidyut K., Mukhopadhyay S. (2021). Target based chemotherapeutic advancement of ruthenium complexes. Coord. Chem. Rev..

[10] Leon I.E., Cadavid-Vargas J.F., di Virgilio A.L., Etcheverry S.B. (2017). Vanadium, Ruthenium and Copper Compounds: A New Class of Nonplatinum Metallodrugs with Anticancer Activity. Curr. Med. Chem..

[11] Lazarević T., Rilak A., Bugarčić Ž.D. (2017). Platinum, palladium, gold and ruthenium complexes as anticancer agents: Current clinical uses, cytotoxicity studies and future perspectives. Eur. J. Med. Chem..

[12] Anthony E.J., Bolitho E.M., Bridgewater H.E., Carter O.W., Donnelly J.M., Imberti C., Lant E.C., Lermyte F., Needham R.J., Palau M. (2020). Metallodrugs are unique: Opportunities and challenges of discovery and development. Chem. Sci..

[13] Kar K., Ghost D., Kabi B., Chandra A. (2022). A concise review on cobalt Schiff base complexes as anticancer agents. Polyhedron.

[14] Sainath B.A., Poonam R.I., Mrunalini K., Makarand V.P., Srushti P., Vasudev B. (2022). Silver Complexes of N-Heterocyclic Carbenes as Anticancer Agents: A Review. Int. J. Life Sci. Pharma Res..

[15] Omondi R.O., Ojwach S.O., Jaganyi D. (2020). Review of comparative studies of cytotoxic activities of Pt(II), Pd(II), Ru(II)/(III) and Au(III) complexes, their kinetics of ligand substitution reactions and DNA/BSA interactions. Inorganica Chim. Acta.

[16] Gasparin C.B., Pilger D.A. (2023). 8-hydroxyquinoline, derivatives and metal-complexes: A review of antileukemia activities. ChemistrySelect.

[17] Frezza M., Hindo S., Chen D., Davenport A., Schmitt S., Tomco D., Ping Dou Q. (2010). Novel Metals and Metal Complexes as Platforms for Cancer Therapy. Curr. Pharm. Des..

[18] Stern B.R., Solioz M., Krewski D., Aggett P., Aw T.C., Baker S., Crump K., Dourson M., Haber L., Hertzberg R. (2007). Copper and Human Health: Biochemistry, Genetics, and Strategies for Modeling Dose-response Relationships. J. Toxicol. Environ. Health B.

[19] Ng C.H., Kong K.C., Von S.T., Balraj P., Jensen P., Thirthagiri E., Hamada H., Chikira M. (2008). Synthesis, characterization, DNA-binding study and anticancer properties of ternary metal(ii) complexes of edda and an intercalating ligand. Dalton Trans..

[20] Balsa L.M., Baran E.J., León I.E. (2022). Copper complexes as antitumor agents: In vitro and in vivo evidences. Curr. Med. Chem..

[21] Denoyer D., Masaldan S., La Fontaine S., Cater M.A. (2015). Targeting copper in cancer therapy: Copper That Cancer. Metallomics.

[22] Santini C., Pellei M., Gandin V., Porchia M., Tisato F., Marzano C. (2014). Advances in Copper Complexes as Anticancer Agents. Chem. Rev..

[23] Ruiz-Azuara L. (1991). Process to Obtain New Mixed Copper Aminoacidate Complexes from Phenylate Phenathrolines to Be Used as Anticancerigenic Agents. European Patent.

[24] Ruiz-Azuara L. (1996). Copper Amino Acidate Diimine Nitrate Compounds and Their Methyl Derivatives and a Process for Preparing Them. U.S. Patent.

[25] Ruiz-Azuara L. (2019). Casiopeina Parenteral Composition and Uses of the Same. Mexican Patent.

[26] De Vizcaya-Ruiz A., Rivero-Muller A., Ruiz-Ramirez L., Kass G.E.N., Kelland L.R., Orr R.M., Dobrota M. (2000). Induction of apoptosis by a novel copper-based anticancer compound, Casiopeina II, in L1210 murine leukaemia and CH1 human ovarian carcinoma cells. Toxicol. In Vitro.

[27] Buchtík R., Trávníček Z., Vančo J., Herchel R., Dvořák Z. (2011). Synthesis, characterization, DNA interaction and cleavage, and in vitro cytotoxicity of copper(ii) mixed-ligand complexes with 2-phenyl-3-hydroxy-4(1H)-quinolinone. Dalton Trans..

[28] Buchtík R., Trávníček Z., Vančo J. (2012). In vitro cytotoxicity, DNA cleavage and SOD-mimic activity of copper(II) mixed-ligand quinolinonato complexes. J. Inorg. Biochem..

[29] Trávníček Z., Vančo J., Hošek J., Buchtík R., Dvořák Z. (2012). Cellular responses induced by Cu(II) quinolinonato complexes in human tumor and hepatic cells. Chem. Cent. J..

[30] Vančo J., Trávníček Z., Hošek J., Dvořák Z. (2022). Heteroleptic copper(II) complexes of prenylated flavonoid osajin behave as selective and effective antiproliferative and anti-inflammatory agents. J. Inorg. Biochem.

[31] Vančo J., Trávníček Z., Hošek J., Malina T., Dvořák Z. (2021). Copper(II) complexes containing natural flavonoid pomiferin show considerable in vitro cytotoxicity and anti-inflammatory effects. Int. J. Mol. Sci..

[32] Gupte A., Mumper R.J. (2009). Elevated copper and oxidative stress in cancer cells as a target for cancer treatment. Cancer Treat. Rev..

[33] Ge E.J., Bush A.I., Casini A., Cobine P.A., Cross J.R., DeNicola G.M., Dou Q.P., Franz K.J., Gohil V.M., Gupta S. (2022). Connecting copper and cancer: From transition metal signalling to metalloplasia. Nat. Rev..

[34] Gul N.S., Khan T.M., Chen M., Huang K.B., Hou C., Choudhary M.I., Liang H., Chen Z.F. (2020). New copper complexes inducing bimodal death through apoptosis and autophagy in A549 cancer cells. J. Inorg. Biochem..

[35] Trejo-Solís C., Jimenez-Farfan D., Rodriguez-Enriquez S., Fernandez-Valverde F., Cruz-Salgado A., Ruiz-Azuara L., Sotelo J. (2012). Copper compound induces autophagy and apoptosis of glioma cells by reactive oxygen species and jnk activation. BMC Cancer.

[36] Marín-Hernández A., Gallardo-Pérez J.C., López-Ramírez S.Y., García-García J.D., Rodríguez-Zavala J.S., Ruiz-Ramírez L., Gracia-Mora I., Zentella-Dehesa A., Sosa-Garrocho M., Macías-Silva M. (2012). Casiopeina II-gly and bromo-pyruvate inhibition of tumor hexokinase, glycolysis, and oxidative phosphorylation. Arch. Toxicol..

[37] Anzenbacher P., Anzenbacherová E. (2001). Cytochromes P450 and metabolism of xenobiotics. Cell Mol. Life Sci..

[38] Grüner B., Brynda J., Das V., Šicha V., Štěpánková J., Nekvinda J., Holub J., Pospisilova K., Fábry M., Pachl P. (2019). Metallacarborane Sulfamides: Unconventional, Specific, and Highly Selective Inhibitors of Carbonic Anhydrase IX. J. Med. Chem..

[39] Borkova L., Frydrych I., Jakubcová N., Adamek R., Lišková B., Gurská S., Medvedíková M., Hajduch M., Urban M. (2020). Synthesis and biological evaluation of triterpenoid thiazoles derived from betulonic acid, dihydrobetulonic acid, and ursonic acid. Eur. J. Med. Chem..

[40] Phillips I., Shephard E., Ortiz De Montellano P. (2013). Cytochrome P450 Protocols.

[41] Kumar Singh J., Olanki A. (2012). Rapid Equilibrium Dialysis (RED): An In-vitro High-Throughput Screening Technique for Plasma Protein Binding using Human and Rat Plasma. J. Bioequivalence Bioavailab..

[42] Di L., Kerns E. (2003). Profiling drug-like properties in discovery research. Curr. Opin. Chem. Biol..

[43] Schenkman J., Jansson I. (2005). Spectral Analyses of Cytochromes P450. Methods Mol. Biol..

[44] (2013). Spartan 14.

[45] Nassar A.F., Hollenberg P.F., Scatina J. (2009). Drug Metabolism Handbook: Concepts and Applications.

[46] Skolnik S., Lin X., Wang J., Chen X., He T., Zhang B. (2010). Towards Prediction of In Vivo Intestinal Absorption Using a 96-Well Caco-2 Assay. J. Pharm. Sci..

[47] Daly A., Rettie A., Fowler D., Miners J. (2018). Pharmacogenomics of CYP2C9: Functional and Clinical Considerations. J. Pers. Med..

[48] Bu H.Z. (2006). A Literature Review of Enzyme Kinetic Parameters for CYP3A4-Mediated Metabolic Reactions of 113 Drugs in Human Liver Microsomes: Structure- Kinetics Relationship Assessment. Curr. Drug. Met..

[49] Hendrychová T., Anzenbacherová E., Hudeček J., Skopalík J., Lange R., Hildebrandt P., Otyepka M., Anzenbacher P. (2011). Flexibility of human cytochrome P450 enzymes: Molecular dynamics and spectroscopy reveal important function-related variations. Biochim. Biophys. Acta.

[50] Campero-Peredo C., Bravo-Gómez M.E., Hernández-Ojeda S.L., del Rosario Olguin-Reyes S., Espinosa-Aguirre J.J., Ruiz-Azuara L. (2016). Effect of [Cu(4,7-dimethyl-1,10-phenanthroline)(acetylacetonato)]NO_3_, Casiopeína III-Ea, on the activity of cytochrome P450. Toxicol. In Vitro.

[51] Jefcoate C.R. (1978). Measurement of substrate and inhibitor binding to microsomal cytochrome P-450 by optical-difference spectroscopy. Methods Enzymol..

[52] Kastritis P., Bonvin A. (2013). On the binding affinity of macromolecular interactions: Daring to ask why proteins interact. J. R. Soc. Interface.

[53] Jelesarov I., Bosshard H.R. (1999). Isothermal titration calorimetry and differential scanning calorimetry as complementary tools to investigate the energetics of biomolecular recognition. J. Mol. Recognit..

