# Supplementary material for: Highly Cytotoxic Copper(II) Mixed-Ligand Quinolinonato Complexes: Pharmacokinetic Properties and Interactions with Drug Metabolizing Cytochromes P450
Source: Pharmaceutics. 2023 Apr 21;15(4):1314. doi: 10.3390/pharmaceutics15041314 (PMC10146558; doi:10.3390/pharmaceutics15041314)
Supplement: Supplementary file 1 [file pharmaceutics-15-01314-s001.zip › pharmaceutics-2281853-supplementary.pdf]

Supplementary Materials

for

Highly cytotoxic copper(II) mixed-ligand quinolinonato complexes: pharmacokinetic properties and interactions with drug metabolizing cytochromes P450

*Martina Medvedíková,[1,2] Václav Ranc[2], Ján Vančo,[3] Zdeněk Trávníček,[3 *] Pavel Anzenbacher[1,2]*

[1] Department of Pharmacology, Faculty of Medicine and Dentistry, Palacký University in Olomouc, Hněvotínská 3, CZ-779 00 Olomouc, Czech Republic.

[2] Institute of Molecular and Translation Medicine, Faculty of Medicine and Dentistry, Palacký University in Olomouc, Hněvotínská 5, CZ-779 00 Olomouc, Czech Republic.

[3] Regional Centre of Advanced Technologies and Materials (RCPTM), Czech Advanced Technology and Research Institute (CATRIN), Palacký University in Olomouc, Šlechtitelů 27, CZ-779 00 Olomouc, Czech Republic.

<u>Keywords:</u> copper (II) complexes, quinolinonato derivatives, cytochrome P450, isothermal titration calorimetry.

Table of Contents	Page
**Table S1:** The coordinates for the optimized geometry of the $[Cu(mphen)(qui)]^+$ complex cation using the ϖB97X-D/LACVP level of theory in the XMol XYZ format.	S3
**Figure S1:** Stability of the tested complexes with plasma **(a)** and PBS **(b)**. The stability was determined as the mean of the two independent experiments performed in triplicates ± SD and is expressed as a percentage of remaining relative to the control (time 0). The studied compounds (final incubation concentration was 10 μM) were incubated with plasma and PBS at seven different time points (0, 30 min, 1, 2, 24, 48, 72 h) at 37°C.	S4
**Figure S2:** The effect of complex **1 (a)**, complex **2 (b)**, free ligand Hqui **(c)** on enzymatic activity of all forms of cytochrome P450. Complementary experiments showing the effect of ligand mphen **(d)** and copper nitrate trihydrate **(e)** on enzymatic activity of CYP2C9, CYP2C19 and CYP3A with the specific substrates (Table 1 in the main text) in HLMs were performed. The inhibition of activity is determined as the mean of the two independent experiments performed in triplicates ± SD and is expressed as a percentage of activity remaining relative to the control (set to 100%, without the addition of the studied compounds). Concentrations of the complexes and ligands in reaction mixtures were 0, 10, 25, 50, 75 and 100 μM. TST = testosterone, MDZ = midazolam.	S5
**Figure S3:** Comparison of IC_{50} **(a)** and Ki **(b)** of the complexes **1** and **2** for inhibition of the selected forms of cytochrome P450.	S5
**Figure S4:** **(a)** Dixon plot for inhibition of CYP3A4/5 (testosterone 6β-hydroxylation) by $Cu(NO_3)_2 \cdot 3H_2O$ and **(c)** for inhibition of CYP3A4 (midazolam 1'-hydroxylation) by $Cu(NO_3)_2 \cdot 3H_2O$, **(b)** Lineweaver – Burk plot for inhibition of CYP3A4/5 (testosterone 6β-hydroxylation) enzymatic activity by $Cu(NO_3)_2 \cdot 3H_2O$ at four substrate concentrations (50, 100, 200, 400 μM) and **(d)** for inhibition of CYP3A4 (midazolam 1´-hydroxylation) by $Cu(NO_3)_2 \cdot 3H_2O$ at four concentrations (1.1, 2.2, 4.4, 8.8 μM) for eight concentrations of $Cu(NO_3)_2 \cdot 3H_2O$ (0, 0.8, 1.6, 3.2, 6.4, 12.8, 25 and 50 μM).	S6
**Figure S5:** Stability of the tested complexes with NADPH generating system. The stability is determined as the mean of the two independent experiments performed in triplicates ± SD and is expressed as a percentage of remaining relative to the control (time 0). The studied compounds (the final incubation concentration was 10 μM) were incubated with NADPH generating system at ten different time points (0, 5, 10, 15, 20, 25, 30, 35, 45 and 60 min.) at 37 °C.	S6
**Figure S6:** Single Point Assay of complexes **1** and **2**, and DMSO as a vehicle solvent in the presence or absence of NADPH. In one case, the tested complexes were preincubated in the presence of NADPH and with microsomes at a concentration 10 times higher than in conventional activity testing. In the second case, NADPH was added to the sample containing microsomes and the compounds only after preincubation.	S7

Table S1. The coordinates for the optimized geometry of the [Cu(mphen)(qui)]$^+$ complex cation using the ϖB97X-D/LACVP level of theory in the XMol XYZ format.

54

00000001

Cu	0.631000	-0.459000	-0.055000
N	2.003000	1.000000	0.015000
N	2.255000	-1.632000	-0.114000
N	-4.370000	-0.147000	0.018000
H	-5.271000	0.308000	-0.018000
O	-0.691000	-1.880000	-0.055000
O	-0.843000	0.741000	-0.070000
C	1.779000	2.310000	0.088000
H	0.740000	2.614000	0.116000
C	2.857000	3.214000	0.129000
H	2.658000	4.275000	0.189000
C	4.157000	2.738000	0.092000
H	4.986000	3.434000	0.122000
C	4.405000	1.345000	0.018000
C	3.273000	0.516000	-0.017000
C	3.406000	-0.906000	-0.087000
C	4.678000	-1.490000	-0.122000
C	5.829000	-0.627000	-0.089000
H	6.811000	-1.087000	-0.118000
C	5.725000	0.736000	-0.022000
C	2.311000	-2.962000	-0.174000
H	1.361000	-3.481000	-0.190000
C	3.549000	-3.631000	-0.213000
H	3.566000	-4.711000	-0.263000
C	4.726000	-2.903000	-0.189000
H	5.685000	-3.408000	-0.219000
C	-1.905000	-1.366000	-0.042000
C	-2.008000	0.052000	-0.047000
C	-3.259000	0.656000	-0.005000
C	-4.336000	-1.521000	0.017000
C	-3.081000	-2.171000	-0.010000
C	-3.038000	-3.586000	-0.010000
H	-2.069000	-4.068000	-0.027000
C	-4.204000	-4.315000	0.012000
H	-4.173000	-5.398000	0.012000
C	-5.456000	-3.654000	0.034000
H	-6.369000	-4.237000	0.050000
C	-5.529000	-2.278000	0.037000
H	-6.489000	-1.773000	0.055000
C	-3.477000	2.113000	0.027000
C	-4.504000	2.658000	0.814000
H	-5.100000	2.018000	1.458000
C	-4.733000	4.033000	0.819000
H	-5.523000	4.445000	1.437000
C	-3.941000	4.876000	0.039000

H	-4.121000	5.945000	0.041000
C	-2.911000	4.340000	-0.738000
H	-2.297000	4.994000	-1.347000
C	-2.674000	2.968000	-0.744000
H	-1.874000	2.547000	-1.339000
C	6.946000	1.616000	0.011000
H	6.968000	2.299000	-0.845000
H	7.858000	1.017000	-0.019000
H	6.976000	2.222000	0.923000

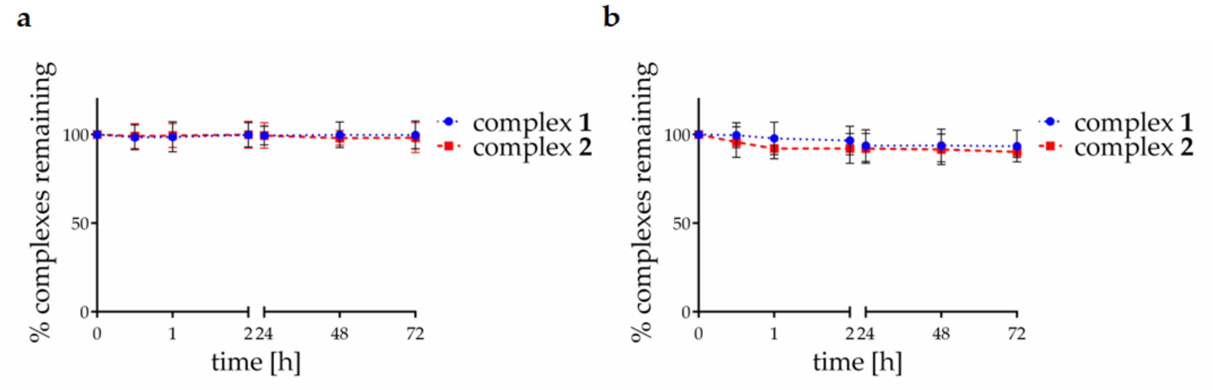

Figure S1. Stability of the tested complexes with plasma **(a)** and PBS **(b)**. The stability was determined as the mean of the two independent experiments performed in triplicates ± SD and is expressed as a percentage of remaining relative to the control (time 0). The studied compounds (final incubation concentration was 10 μM) were incubated with plasma and PBS at seven different time points (0, 30 min, 1, 2, 24, 48, 72 h) at 37°C.

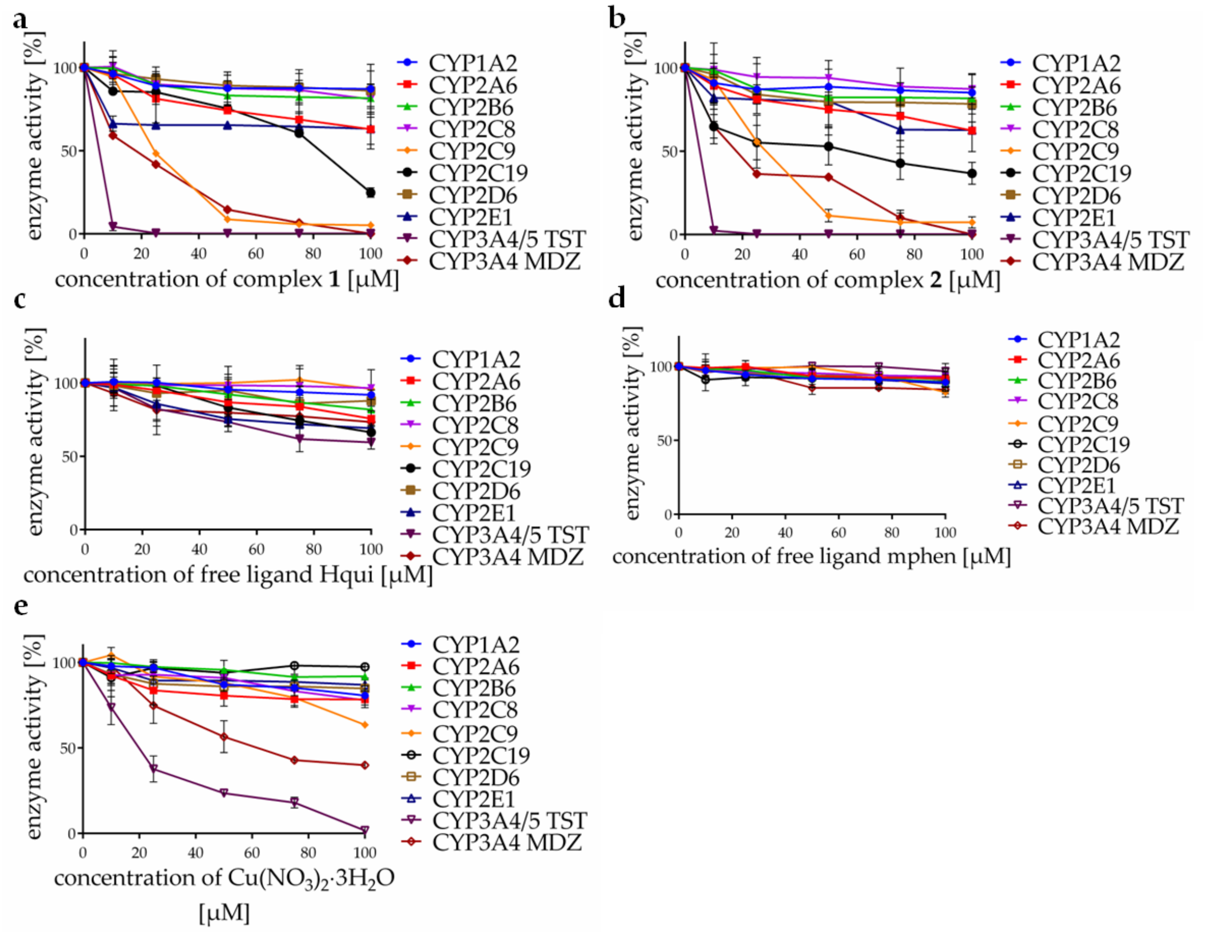

Figure S2. The effect of complex **1** **(a)**, complex **2** **(b)**, free ligand Hqui **(c)** on enzymatic activity of all forms of cytochrome P450. Complementary experiments showing the effect of ligand mphen **(d)** and copper nitrate trihydrate **(e)** on enzymatic activity of CYP2C9, CYP2C19 and CYP3A with the specific substrates (Table 1 in the main text) in HLMs were performed. The inhibition of activity is determined as the mean of the two independent experiments performed in triplicates ± SD and is expressed as a percentage of activity remaining relative to the control (set to 100%, without the addition of the studied compounds). Concentrations of the complexes and ligands in reaction mixtures were 0, 10, 25, 50, 75 and 100 μM. TST = testosterone, MDZ = midazolam.

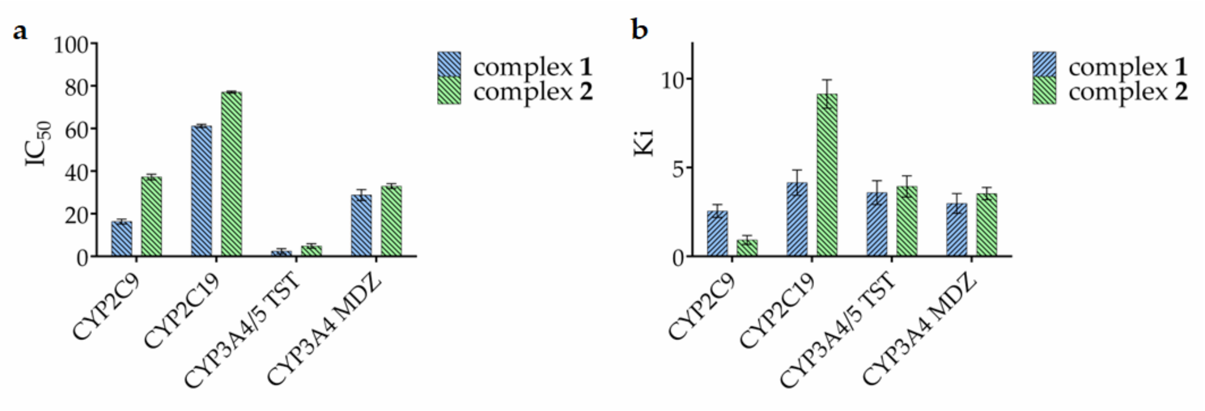

Figure S3. Comparison of IC_{50} **(a)** and Ki **(b)** of the complexes **1** and **2** for inhibition of the selected forms of cytochrome P450.

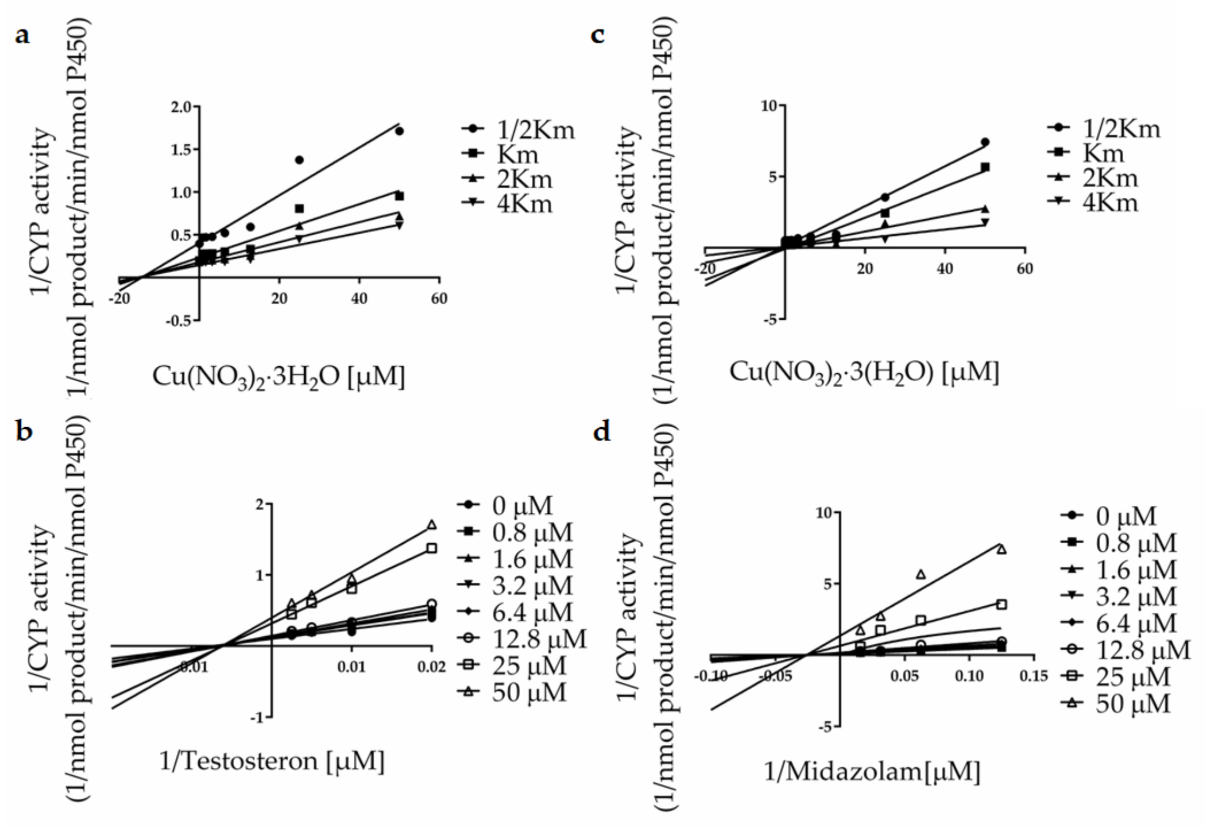

Figure S4. (a) Dixon plot for inhibition of CYP3A4/5 (testosterone 6β-hydroxylation) by $Cu(NO_3)_2{\cdot}3H_2O$ and **(c)** for inhibition of CYP3A4 (midazolam 1'-hydroxylation) by $Cu(NO_3)_2{\cdot}3H_2O$, **(b)** Lineweaver – Burk plot for inhibition of CYP3A4/5 (testosterone 6β-hydroxylation) enzymatic activity by $Cu(NO_3)_2{\cdot}3H_2O$ at four substrate concentrations (50, 100, 200, 400 μM) and **(d)** for inhibition of CYP3A4 (midazolam 1´-hydroxylation) by $Cu(NO_3)_2{\cdot}3H_2O$ at four concentrations (1.1, 2.2, 4.4, 8.8 μM) for eight concentrations of $Cu(NO_3)_2{\cdot}3H_2O$ (0, 0.8, 1.6, 3.2, 6.4, 12.8, 25 and 50 μM).

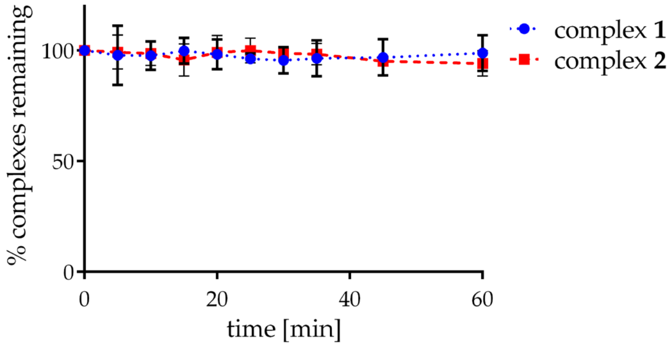

Figure S5. Stability of the tested complexes with NADPH generating system. The stability is determined as the mean of the two independent experiments performed in triplicates ± SD and is expressed as a percentage of remaining relative to the control (time 0). The studied compounds (the final incubation concentration was 10 μM) were incubated with NADPH generating system at ten different time points (0, 5, 10, 15, 20, 25, 30, 35, 45 and 60 min.) at 37 °C.

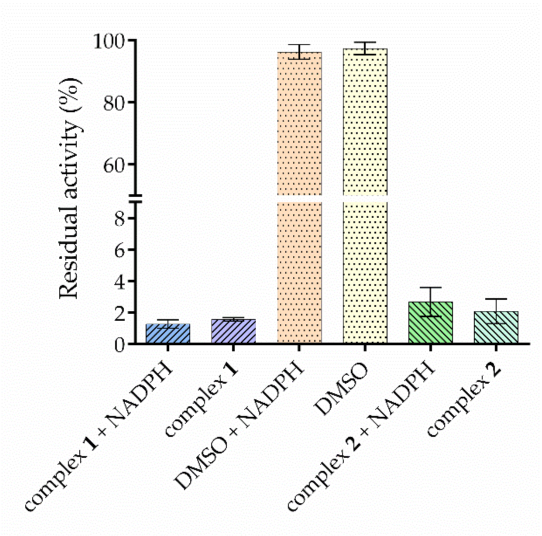

Figure S6. Single Point Assay of complexes **1** and **2**, and DMSO as a vehicle solvent in the presence or absence of NADPH. In one case, the tested complexes were preincubated in the presence of NADPH and with microsomes at a concentration 10 times higher than in conventional activity testing. In the second case, NADPH was added to the sample containing microsomes and the compounds only after preincubation.
